# Comparative Detection and Genetic Characterization of Feline Panleukopenia Virus in Bangladesh

**DOI:** 10.1002/vms3.70594

**Published:** 2025-08-22

**Authors:** Nurejunnati Jeba, Roni Mia, Md. Masum Billah Tarafder, Anandha Mozumder, Raduyan Farazi, S. M. Nazmul Hasan, Mohammad Bayazid Bostami, A. K. M. Anisur Rahman, Abdul Mannan, Sharmin Akter, Sukumar Saha, Tofazzal Islam, Elcio Leal, Antonio Charlys da Costa, Md. Golzar Hossain

**Affiliations:** ^1^ Department of Microbiology and Hygiene Bangladesh Agricultural University Mymensingh Bangladesh; ^2^ Teaching and Training Pet Hospital and Research Center Chattogram Veterinary and Animal Sciences University Chattogram Bangladesh; ^3^ Department of Medicine Bangladesh Agricultural University Mymensingh Bangladesh; ^4^ Department of Physiology Bangladesh Agricultural University Mymensingh Bangladesh; ^5^ Institute of Biotechnology and Genetic Engineering Gazipur Agricultural University Gazipur Bangladesh; ^6^ Institute of Biological Sciences Federal University of Pará Belem Pará Brazil; ^7^ Instituto de Medicina Tropical Faculdade de Medicina Universidade de São Paulo São Paulo, State of São Paulo Brazil

**Keywords:** *Feline panleukopenia* virus, IC strip test, PCR, phylogenetic analysis, VP2 gene mutations

## Abstract

**Background:**

*Feline panleukopenia* virus (FPV) is a highly contagious and often fatal disease affecting domestic and wild felines. Accurate diagnosis and understanding of circulating strains are essential for effective control.

**Objectives:**

This study aimed to evaluate the diagnostic accuracy of a rapid immunochromatographic (IC) antigen test compared to PCR for FPV detection in clinically suspected pet cats in Bangladesh. It also aimed to investigate the genetic and evolutionary characteristics of circulating FPV strains.

**Methods:**

Faecal or rectal swab samples from suspected cats were tested using both IC strip tests and PCR. Sensitivity and specificity of the IC test were analysed using PCR as the reference. Partial sequencing of the *VP2* gene was performed on four PCR‐positive samples for phylogenetic and mutational analysis. Structural modelling of VP2 proteins was conducted to predict conformational changes.

**Results:**

The IC test detected FPV in 84% of cases, whereas PCR confirmed only 60%, indicating a 24% false‐positive rate. PCR showed higher diagnostic reliability. FPV prevalence was 92% among unvaccinated cats. Phylogenetic analysis of VP2 sequences revealed close genetic similarity with Chinese and Portuguese strains, suggesting possible cross‐border transmission. Mutations such as A756G, A896G, E299G and T236I were consistently observed. Structural modelling indicated minor conformational changes in VP2.

**Conclusion and clinical significance:**

PCR offers superior accuracy over IC testing for FPV diagnosis. Mutational changes may impact antigenicity and diagnostic performance. Improved diagnostic accuracy, molecular surveillance and updated vaccination strategies are essential to control FPV outbreaks in feline populations.

## Graphical abstract

PCR analysis of rectal swab samples from cats suspected of *Feline panleukopenia* virus (FPV) infection demonstrated higher diagnostic reliability compared to the immunochromatographic (IC) strip test. Epidemiological and demographic data revealed a high prevalence of FPV among unvaccinated, male and local‐breed cats. Foul‐smelling faeces and diarrhoea were identified as key clinical indicators. Phylogenetic analysis of *VP2 gene* sequences showed close genetic similarity to strains from China and Portugal. Several amino acid mutations were detected in the VP2 proteins, which may cause minor conformational changes in the protein structure.

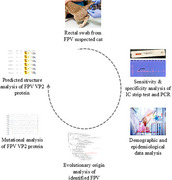

## 1 Introduction

*Feline panleukopenia* virus (FPV) is a highly contagious and significant viral pathogen affecting domestic cats, often leading to fatal outcomes in severe cases (Barrs 2019; Kruse et al. 2010). It infects all felids and some related species, including domestic and wild cats, raccoons and minks (Barrs 2019). FPV primarily causes high mortality in kittens with underdeveloped immune systems (Cave et al. 2002; Raheena et al. 2017). Transmission occurs mainly through direct contact with infected cats, respiratory droplets and contaminated fomites (Truyen et al. 2009). The severity of FPV infection is closely related to clinical signs and symptoms (Porporato et al. 2018), which vary based on age, immune status, vaccination history and concurrent infections (Foley et al. 1999; Kruse et al. 2010). Clinical manifestations range from mild illness to severe disease, which can lead to rapid death (Kruse et al. 2010). Infected cats commonly exhibit symptoms such as depression, anorexia, vomiting, diarrhoea and severe dehydration (Sykes 2013). The disease progresses through four clinical forms: subacute, peracute, acute and perinatal. Moreover, the morbidity and mortality rates are influenced by the pathogenicity of the viral strain (Tuzio 2021).

FPV is a small, non‐enveloped, single‐stranded DNA virus belonging to the Parvoviridae family. It shares genetic, structural and antigenic similarities with canine parvovirus (CPV), which can also infect cats (CPV strains 2a, 2b and 2c) (Holzworth 1987; Porporato et al. 2018). The most widely accepted theory suggests that FPV is the ancestral virus of CPV, emerging due to spontaneous genetic mutations (Horiuchi et al. 1998). Feline parvoviruses within the genus Parvovirus include FPV, CPV and mink enteritis virus (MEV), which are closely related antigenically (Carmichael et al. 1980; Horiuchi et al. 1998; Johnson and Spradbrow 1979; Siegl et al. 1985).

The FPV genome is approximately 5.2 kb long and contains four open reading frames (ORFs) encoding two structural proteins (VP1, VP2) and two non‐structural proteins (NS1, NS2) (Balboni et al. 2018; Yang et al. 2022). VP2, encoded by the fourth ORF, constitutes about 90% of the viral capsid. It plays a crucial role in determining host range and contains several B‐cell epitopes responsible for inducing protective antibodies with neutralizing capacity (Chang et al. 2020). Due to its genetic and epidemiological significance, the ORF encoding VP2 has been extensively studied, particularly as an antigen for subunit vaccine development (Chang et al. 2020; Su et al. 2009).

Meta‐analysis data indicate a high global prevalence of FPV infection in domestic cats (Alessa et al. 2026). Several diagnostic methods are available for detecting FPV, including immunochromatographic (IC) strip tests, haemagglutination (HA) tests, serum neutralization (SN) tests, haemagglutination inhibition (HI) assays, SNAP ELISA and PCR (Brower et al. 2004; Jacobson et al. 2021; Raheena et al. 2017). Although FPV does not directly impact the economy, it can cause significant financial losses for breeders and pet owners.

In Bangladesh, a large number of cats die each year due to FPV infection (Kabir et al. 2023). False‐negative or false‐positive test results can occur, making early and accurate diagnosis essential for saving lives. Additionally, significant global variations in FPV infection rates suggest that current preventive measures may be inadequate. Findings emphasize the need for stricter control strategies and a greater focus on risk factors (Alessa et al. 2026). Furthermore, the potential genomic relationship between viral antigenicity and diagnostic test failures should be confirmed using PCR (Jacobson et al. 2021).

As no molecular data on circulating FPV strains have been reported to date from districts of Bangladesh with relatively high pet populations—such as Narayanganj, Narsingdi and Cumilla—this study aims to detect FPV infection in suspected cats using a commercially available rapid antigen detection kit and PCR, followed by sensitivity and specificity analyses. Additionally, the study seeks to characterize the partial *VP2* gene from these underrepresented regions to explore their evolutionary origins. Furthermore, amino acid (aa) mutations and structural alterations in VP2 proteins were examined.

## 2 Methods

### 2.1 Questionnaire Preparation

A structured questionnaire was developed to document the clinico‐epidemiological data of suspected pet cats. It included qualitative data such as age, sex and vaccination history, along with clinical data, including anorexia, vomiting, diarrhoea, foul‐smelling faeces, fever and other symptoms. As the primary aim of this study was the molecular detection and genetic characterization of FPV, information on treatment was not collected during clinical data collection. The questionnaire was designed on the basis of literature reviews relevant to FPV infection. Informed consent was obtained from all cat owners before data and sample collection.

### 2.2 Sample Collection

**FIGURE 1:**
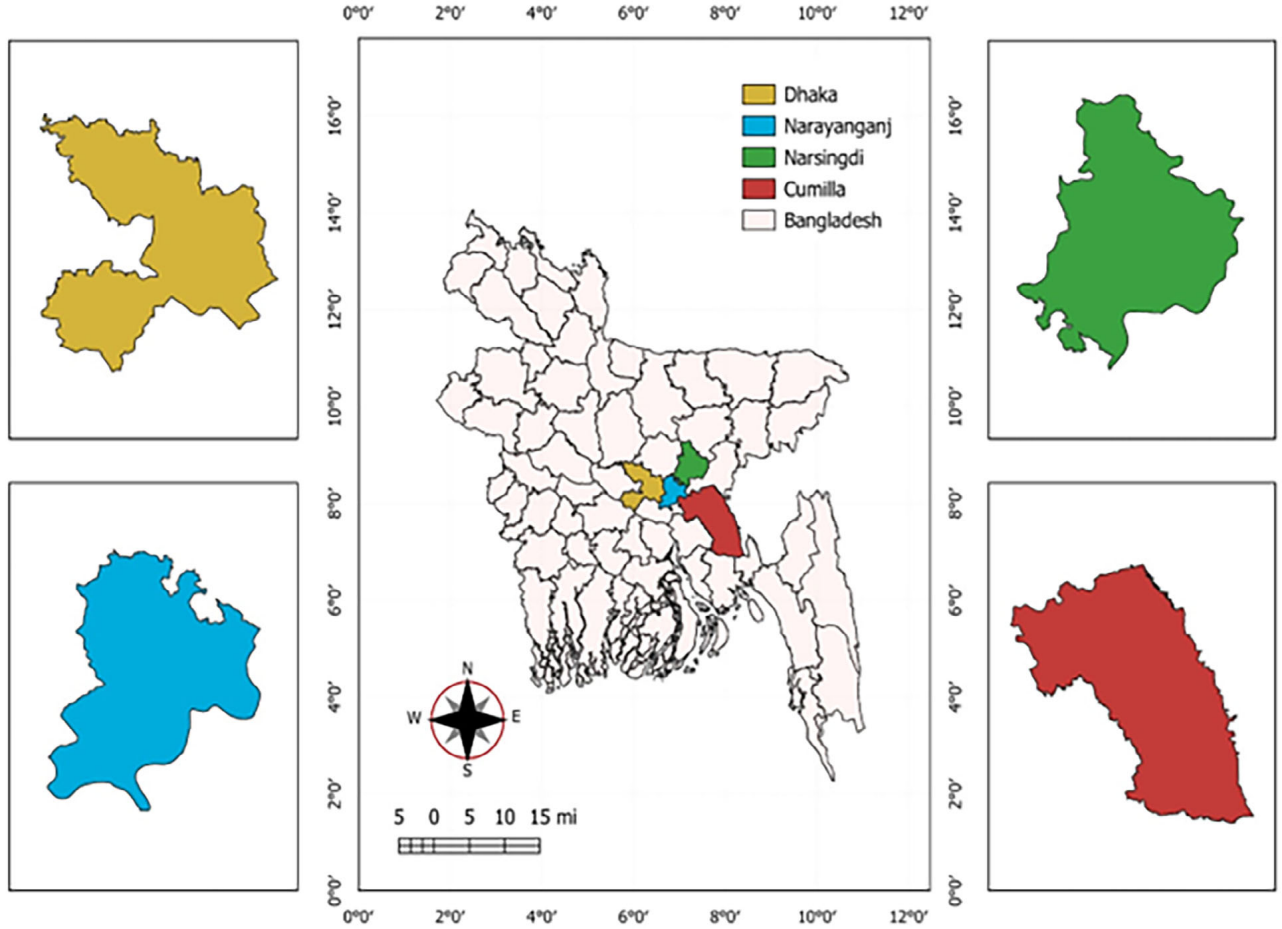
**Geographical representation of the locations included in this FPV study**. The map was created using QGIS software.

The samples were collected from the Teaching and Training Pet Hospital and Research Centre (TTPHRC), Purbachal, a major healthcare facility for pet cats in Dhaka, Bangladesh. The hospital was selected due to its high case volume, providing a representative sample of suspected cases. A convenience sampling approach was used, and pet cats were selected on the basis of their clinical presentation and suspected illness. A total of 50 faecal or rectal swab samples were collected under aseptic conditions. Samples were obtained from four districts: Dhaka, Narayanganj, Narsingdi and Cumilla (Figure 1). The collected samples were transported to the Virology Laboratory at the Department of Microbiology and Hygiene, Bangladesh Agricultural University, and stored at −80°C until further analysis. However, the survival outcomes of cats after diagnosis could not be recorded due to the lack of systematic follow‐up and limited access to hospital records. Similarly, treatment modalities administered to the infected cats were not documented, as they were beyond the scope of the study.

### 2.3 IC Strip Test

**FIGURE 2:**
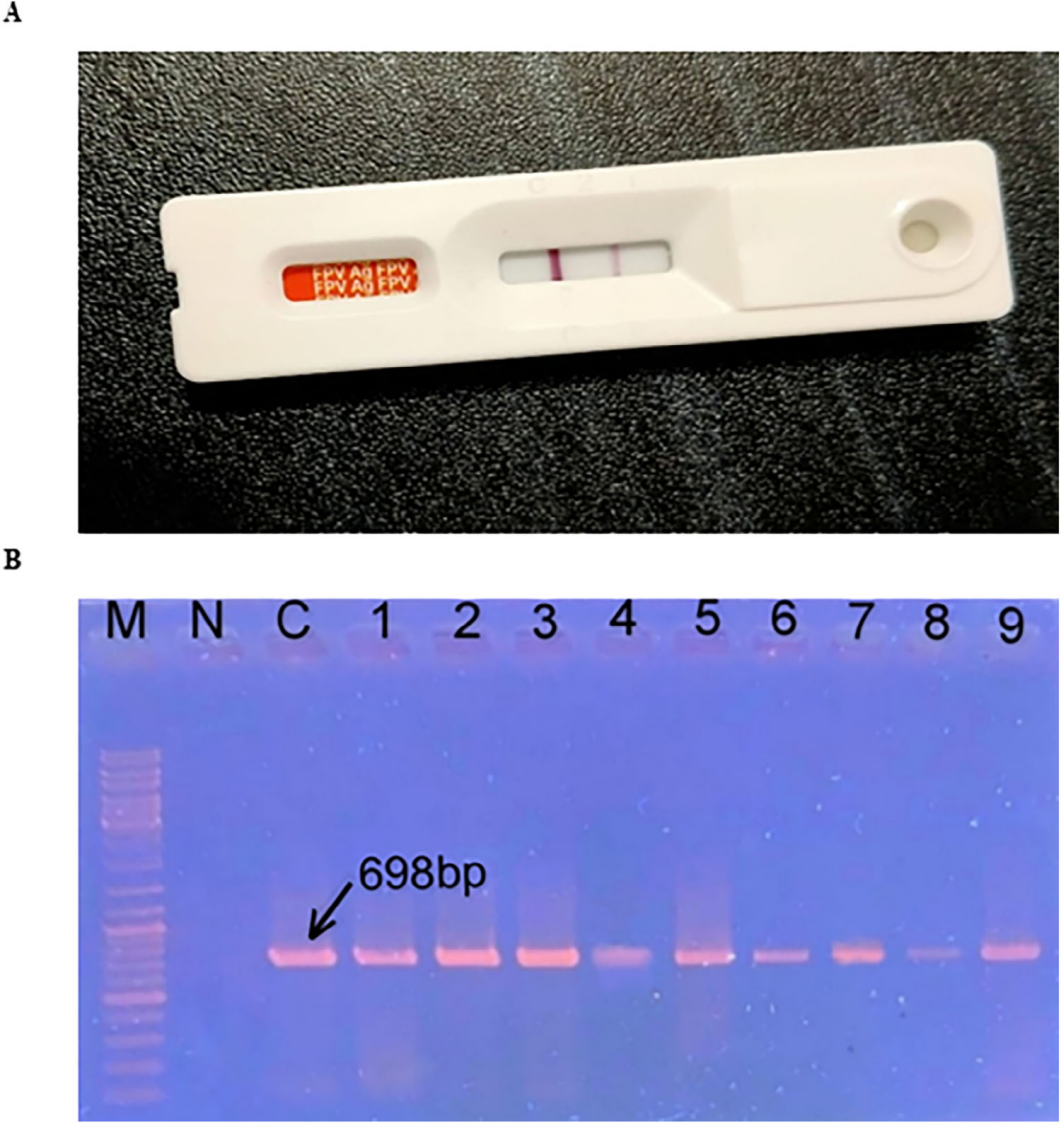
**Identification of FPV**. (A) Preliminary detection using the IC test. Coloured lines in both the test (T) and control (C) regions indicate the presence of the target analyte in the sample, confirming a positive result. (B) Identification by PCR. Gel electrophoresis showing the *VP2* gene amplicons of FPV (698 bp). Lanes: M—100 bp DNA ladder (Thermo Fisher, USA), N—Negative control, C—Positive control, Lanes 1–9—Representative FPV‐positive samples showing bands at approximately 698 bp. FPV, *Feline panleukopenia* virus.

The Rapid FPV Antigen Test Kit (Testsealabs *F. Panleukopenia* Antigen FPV Ag Test, Hangzhou Testsea Biotechnology Co. Ltd.) was used for the preliminary detection of FPV infection. A sufficient amount of the faecal swab sample was mixed with the sample dilution buffer and stirred to disperse the sample. Then, 3–4 drops of the diluted sample were added drop by drop into the test device's sample well. The test was performed following the manufacturer's instructions, and results were interpreted visually on the basis of the presence of coloured lines in the control (C) and test (T) regions. The outcomes were recorded as either positive or negative (Figure 2A).

### 2.4 Extraction of Viral DNA

An adequate amount of PBS was added to the samples and vortexed thoroughly. The samples were then centrifuged at 3000 rpm for 3 min. The clear supernatant was collected and used for DNA extraction. Viral DNA was extracted using the boiling snap‐chilling method, as described previously (Parthiban et al. 2012). Briefly, approximately 200 µL of the prepared sample was boiled for 10 min, followed by rapid cooling on ice for another 10 min. The chilled samples were centrifuged at 10,000 rpm for 10 min, and the clear supernatant was collected as extracted DNA, which was subsequently stored at −80°C for further analysis.

### 2.5 Molecular Detection

A *VP2* gene‐specific primer set (FM‐F: 5′‐GCTTTAGATGATACTCATGT‐3′ and FM‐R: 5′‐GTAGCTTCAGTAATATAGTC‐3′) was used to amplify a 698 bp fragment (Mochizuki et al. 1993; Yang et al. 2010). PCR amplification was performed under the following conditions: initial denaturation at 94°C for 30 s, followed by 30 cycles of denaturation at 94°C for 30 s, annealing at 55°C for 2 min, extension at 72°C for 2 min and a final extension at 72°C for 7 min. The reactions were carried out using GoTaq G2 Green Master Mix (Promega Fisher Scientific, USA). The PCR‐amplified products were separated on a 1.5% agarose gel in Tris‐acetate EDTA buffer, stained with ethidium bromide and electrophoresed for 25 min at 100 V. The amplified products were visualized using a gel documentation system (GelDoc Go, BioRad, USA).

### 2.6 Sensitivity and Specificity Analysis

The sensitivity and specificity of the rapid antigen test (IC strip) and PCR were calculated and expressed as percentages using the following formulas, $\mathrm{sensitivity}\;=\frac{\mathrm{TP}}{\mathrm{TP}+\mathrm{FN}}\;$× 100 and $\mathrm{specificity}\;=\frac{\mathrm{TN}}{\mathrm{TN}+\mathrm{FP}}\;$× 100 as described previously (Baratloo et al. 2015).

### 2.7 Statistical Analysis of Demographic and Epidemiological Data

All collected data were initially organized using Microsoft Excel 2016 and then exported to SPSS for descriptive statistical analysis. Demographic variables such as age, sex, vaccination status and breed were analysed using Pearson's Chi‐square test to determine potential risk factors for FPV infection (Awad et al. 2018). Additionally, multivariable logistic regression analysis (MLRA) was conducted to examine the relationship between clinical signs and FPV infection (Table S1) (Rehme et al. 2022).

### 2.8 Sequencing and Evolutionary Origin Analysis

Four representative PCR‐amplified partial *VP2* gene sequences were selected for sequencing; three FPV‐positive samples containing a 698 bp *VP2* gene fragment, one FPV‐positive sample containing a 1073 bp *VP2* gene fragment. The sequencing was conducted at the National Institute of Bangladesh (https://nib.gov.bd/) and the University of São Paulo, Brazil (Table 1). The raw sequences were edited, annotated and analysed using CLC Sequence Viewer version 8.0 (http://www.clcbio.com) and compared with the reference sequence (MZ712026.1). Additional reference FPV *VP2* gene sequences were retrieved from GenBank for evolutionary analysis. A phylogenetic tree was constructed using MEGA11 software. Nucleotide sequences were aligned using the ClustalW technique. The Tamura–Nei model and Neighbour‐Joining method were used to infer evolutionary relationships (Tamura and Nei 1993). The proportion of trees where associated taxa clustered together is shown below the branches. Initial trees for heuristic searches were automatically generated using the Neighbour‐Joining and BioNJ algorithms on the basis of a pairwise distance matrix computed with the Tamura–Nei model. The topology with the highest log‐likelihood value was selected (Tamura et al. 2021).

**TABLE 1:** List of primers used for sequencing the partial *VP2* gene.

Primer names	Sequence (5′–3′)
FM‐F	GCTTTAGATGATACTCATGT
FM‐R	GTAGCTTCAGTAATATAGTC
FPV_5_F	CCAACCATACCAACTCCATGG
FPV_5a_F	GTTCAACAAGATAAAAGACGTGG
FPV_5_R	CATTAATACTCATTTGTTGAATTGG
FPV_6_F	ATTGCTACCAACAGATCCAATTGGAG
FPV_6a_F	CAAAATATTAACTTTAACCTTCC
FPV_6_R	ACTATGATCTAAATGTTCTTCTAT
FPV_7_F	ATAGAAGAACATTTAGATCATAGT

Abbreviation: FPV, *Feline panleukopenia* virus.

### 2.9 Mutational Analysis and Structural Modelling of VP2 Protein

The identified partial *VP2* gene sequences were compared against the reference sequence (MZ712026.1) and the Indian FPV strain (PP035817.1) for mutational analysis. Using CLC Sequence Viewer version 8.0, the nucleotide sequences were translated into aa sequences and aligned with the reference sequences. For structural analysis, the secondary structures of VP2 proteins were predicted using the SOPMA online server with default parameters. Tertiary structures of the mutant proteins were modelled using SWISS‐MODEL (https://swissmodel.expasy.org/) (Waterhouse et al. 2018). The protein sequence was submitted to the server for template search, and models were selected on the basis of GMQE (Global Model Quality Estimation) scores, query coverage and identity values. The final 3D structural models were visualized using PyMOL software. To validate the modelled structures, Ramachandran plot analysis and *Z*‐score calculations were performed using Procheck tools from SAVESv6.0 (https://saves.mbi.ucla.edu/) (Laskowski et al. 1993) and ProSA‐web (https://prosa.services.came.sbg.ac.at/prosa.php) (Wiederstein and Sippl 2007).

## 3 Results

### 3.1 Comparison of Rapid Antigen Test and PCR for FPV Detection

Initially, FPV was detected using a rapid antigen detection kit from faecal or rectal swab samples of suspected cats, followed by conventional PCR. The results indicated that 84% of the samples (42/50) tested positive using the IC strip test kit (Figure 2A). PCR is considered the gold standard for FPV detection (Abdelbaky et al. 2024). In this study, conventional PCR targeting the *VP2* gene revealed that 60% (30/50) of the samples were positive for FPV (Figure 2B and Table S1). These 30 PCR‐positive samples also tested positive using the IC strip test and were considered true positives. Conversely, 24% (12/50) of the IC test‐positive samples tested negative using PCR and were classified as false positives. These findings suggest that PCR is highly specific compared to the IC strip test for FPV detection.

### 3.2 Epidemiology and Susceptibility Patterns of FPV‐Infected Cats

**FIGURE 3:**
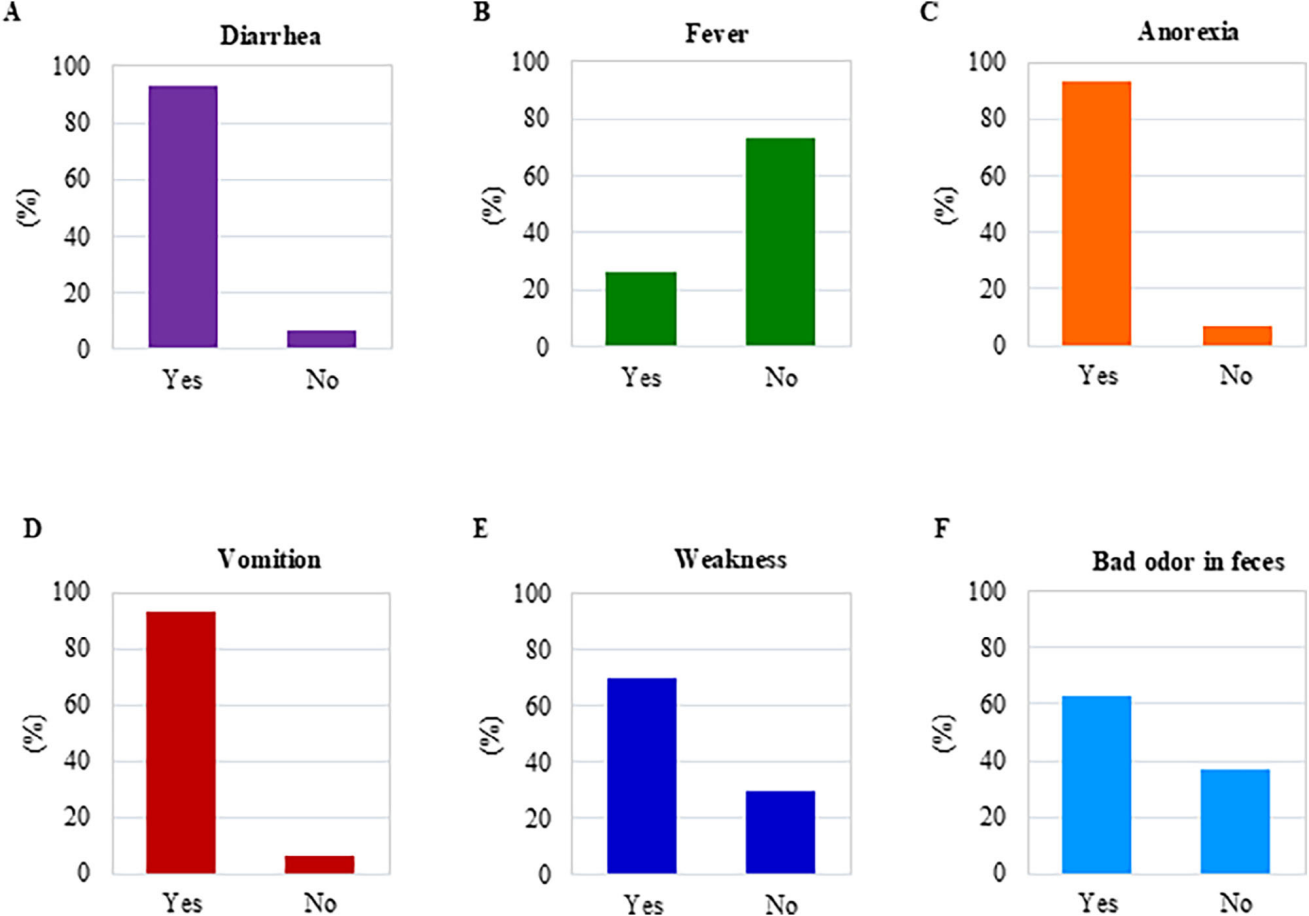
**Distribution of the cat population based on clinical signs and symptoms**. The clustered column bar diagrams illustrate the number of FPV‐infected cats exhibiting clinical symptoms such as diarrhoea, fever, weakness, vomiting, anorexia and foul‐smelling faeces.

**FIGURE 4:**
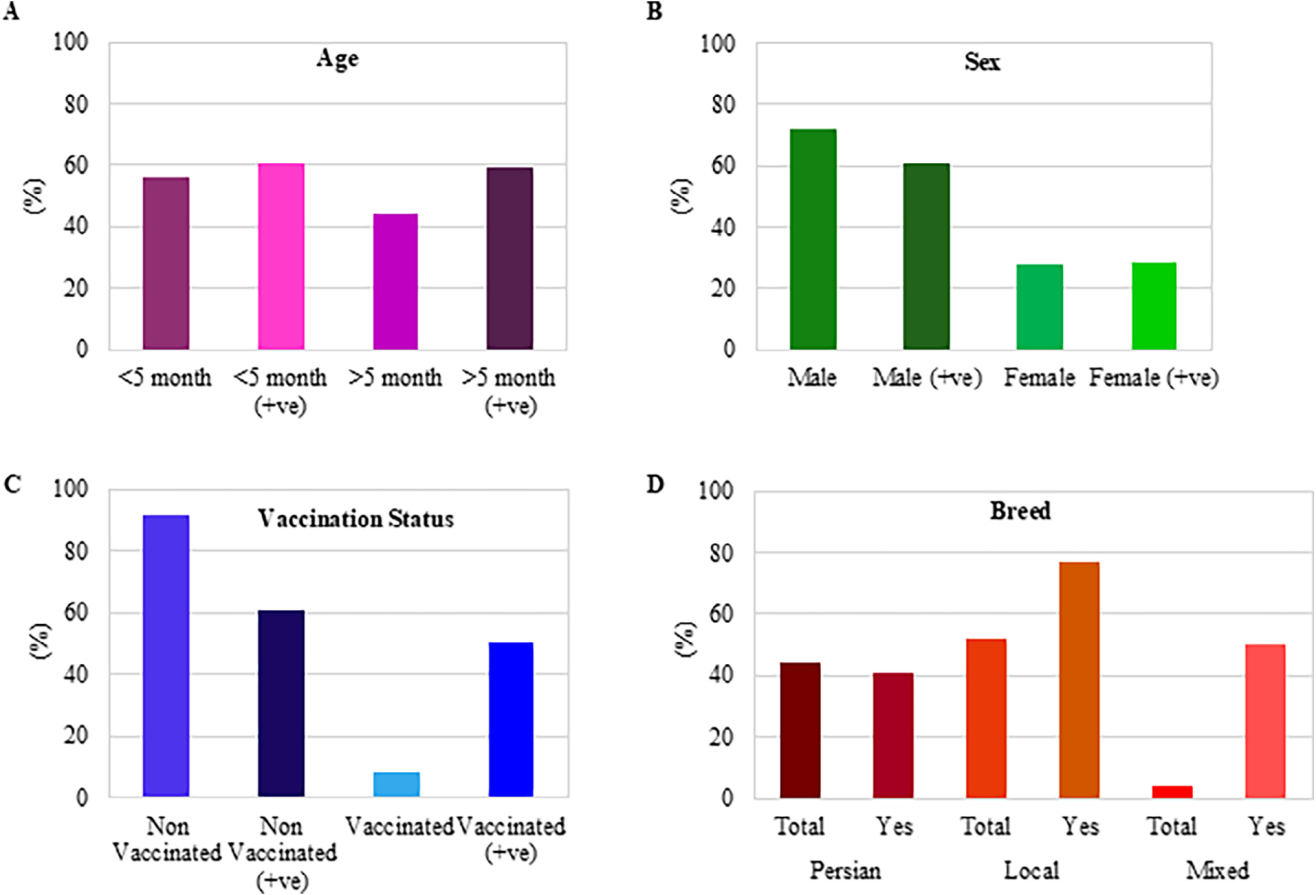
**Distribution of FPV‐infected cats by age, sex and vaccination status**. (A) Age distribution: cats aged ≤5 months versus >5 months. (B) Sex distribution: male versus female cats. (C) Vaccination status: vaccinated versus unvaccinated cats. (D) Breed distribution: Persian, local and mixed breeds.

Most affected cats exhibited common clinical symptoms, including diarrhoea, vomiting, anorexia, foul‐smelling faeces, weakness and elevated body temperature (Table S1 and Figure 3). The ages of the suspected cats ranged from 1 to 84 months, with the majority being male (72%) and a smaller proportion female (28%). A total of 92% (46/50) of the suspected cats were unvaccinated, whereas 8% (4/50) were vaccinated (Table S1 and Figure 4B,C). According to Pearson's Chi‐Square test analysis of positive samples identified by both IC strip and PCR, approximately 93.33% of FPV‐infected cats exhibited diarrhoea, vomiting and anorexia. Additionally, the percentages of FPV‐infected cats displaying fever, weakness and foul‐smelling faeces were 26.67%, 70% and 63.33%, respectively (Figure 3). The study revealed that the susceptibility of kittens (≤5 months) and older cats (>5 months) was similar, with infection rates of 60.71% and 59.09%, respectively (Figure 4A). Furthermore, male cats were found to be more susceptible (73.3%) than female cats (66.7%) (Figure 4B). Among the infected cats, 60.9% were unvaccinated, whereas 50% of the vaccinated cats were also infected (Figure 4C). The highest number of positive cases (76.92%) were found in local breeds, followed by 50% in mixed breeds and 40.9% in Persian breeds (Figure 4D). The study identified diarrhoea and foul‐smelling faeces as diagnostic symptoms of FPV infection. An MLRA showed significant correlations with FPV infection (*p* < 0.05) among suspected cats (Table 2). According to the analysis, cats with diarrhoea (OR = 8.2) and foul‐smelling faeces (OR = 23.1) had 8.2 and 23.1 times higher chances of testing positive for FPV infection, respectively, compared to cats without these symptoms.

**TABLE 2:** Summary of multivariate logistic regression analysis.

	Multivariate	
Variable	OR	*p* value
Diarrhoea	8.2	0.03114
Bad odour in faeces	23.1	0.00536

### 3.3 Sequencing and Evolutionary Origin Analysis

**FIGURE 5:**
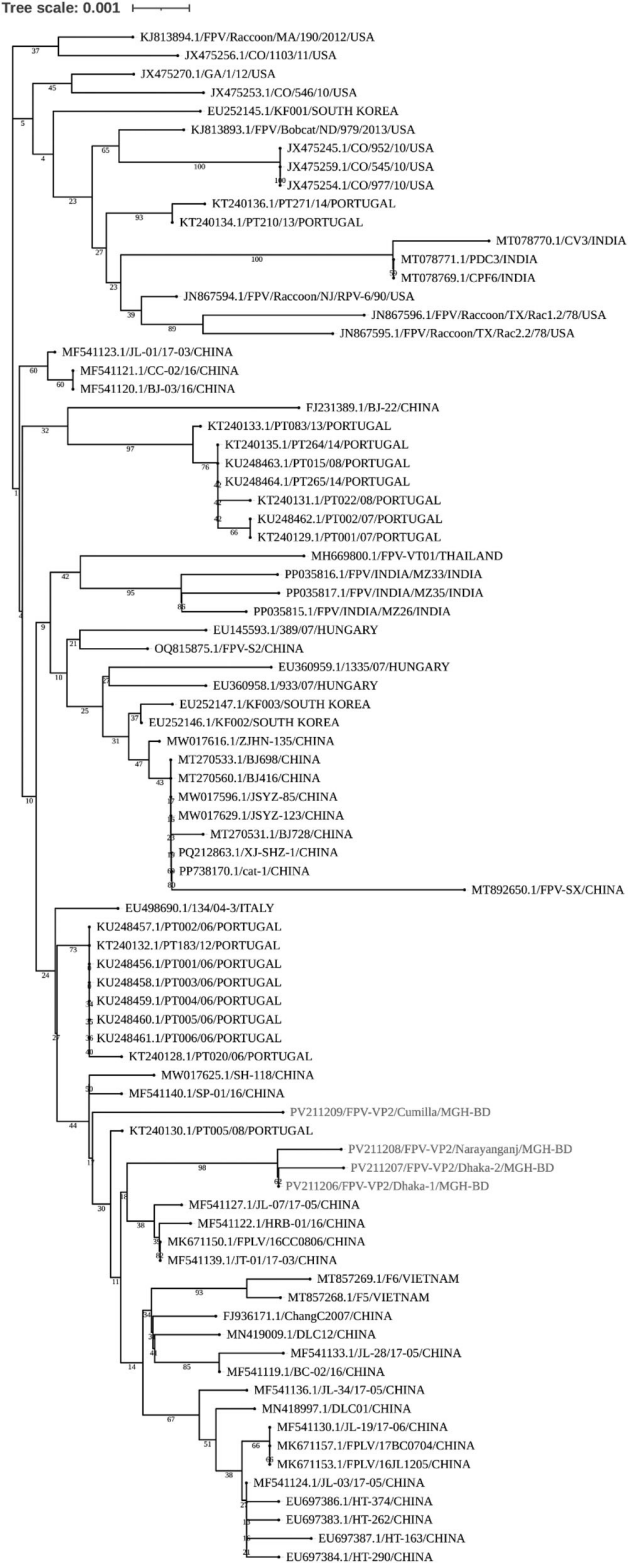
**Phylogenetic tree of FPV isolates identified in this study**. (A) Neighbour‐Joining tree was constructed using 79 partial *VP2* gene sequences retrieved from GenBank. Evolutionary distances were calculated using the Tamura–Nei method. Sequence alignment and evolutionary analyses were performed using MEGA11 software and ClustalW. Sequences obtained in this study are highlighted in blue.

Four representative samples were selected on the basis of PCR band intensity for further genome sequence analysis. The partial *VP2* genes were successfully sequenced using *VP2* gene‐specific primer sets (Table 1). The lengths of the partially sequenced *VP2* genes were 331–969, 325–969, 344–967 and 682–1755 bp, respectively. We obtained accession numbers from GenBank for the identified isolates: PV211206 (FPV‐VP2/Dhaka‐1/MGH‐BD), PV211207 (FPV‐VP2/Dhaka‐2/MGH‐BD), PV211208 (FPV‐VP2/Narayanganj/MGH‐BD) and PV211209 (FPV‐VP2/Cumilla/MGH‐BD). Phylogenetic tree analysis showed that three strains, FPV‐VP2/Dhaka‐1/MGH‐BD, FPV‐VP2/Dhaka‐2/MGH‐BD and FPV‐VP2/Narayanganj/MGH‐BD formed an independent branch and were evolutionarily related to FPV isolates from China. Meanwhile, one strain, FPV‐VP2/Cumilla/MGH‐BD, showed close relation to isolates from both China and Portugal (Figure 5).

### 3.4 Genomic Characteristics of the Identified FPV VP2 Sequences

**FIGURE 6:**
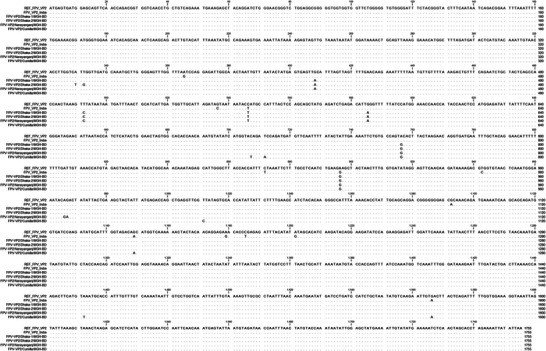
**Nucleotide substitution analysis of the *VP2* gene in identified FPV isolates**. Mutational analysis was conducted by aligning *VP2* gene sequences from this study with reference sequences using CLC Sequence Viewer 8.0. Dots represent identical residues, whereas letters indicate nucleotide substitutions.

The VP2 protein plays a crucial role in determining the host range of the virus and is considered the major capsid protein of parvovirus (Brindhalakshmi et al. 2016). Variations among different parvovirus strains result from mutations in specific aas of the VP2 capsid protein (Chowdhury et al. 2021). Nucleotide substitution analysis, compared to two reference sequences, REF_FPV_VP2 (MZ712026.1) and FPV_VP2_India (PP035817.1), revealed several nucleotide substitutions in all four identified and sequenced isolates (Figure 6). Notably, an A756G substitution was found in all identified strains compared to both reference sequences. Additionally, an A896G substitution was observed in all isolates compared to REF_FPV_VP2, whereas T871C and C942T mutations were reported in all isolates compared to FPV_VP2_India (Figure 6).

### 3.5 AA Mutations and Structural Alterations in VP2 Proteins

**FIGURE 7:**
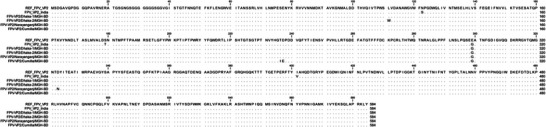
**Amino acid variations in the VP2 protein of identified FPV isolates**. Amino acid sequences of VP2 proteins were aligned and compared with reference strains. Dots indicate identical residues, whereas letters denote substitutions. Analysis was performed using CLC Sequence Viewer 8.0.

**FIGURE 8:**
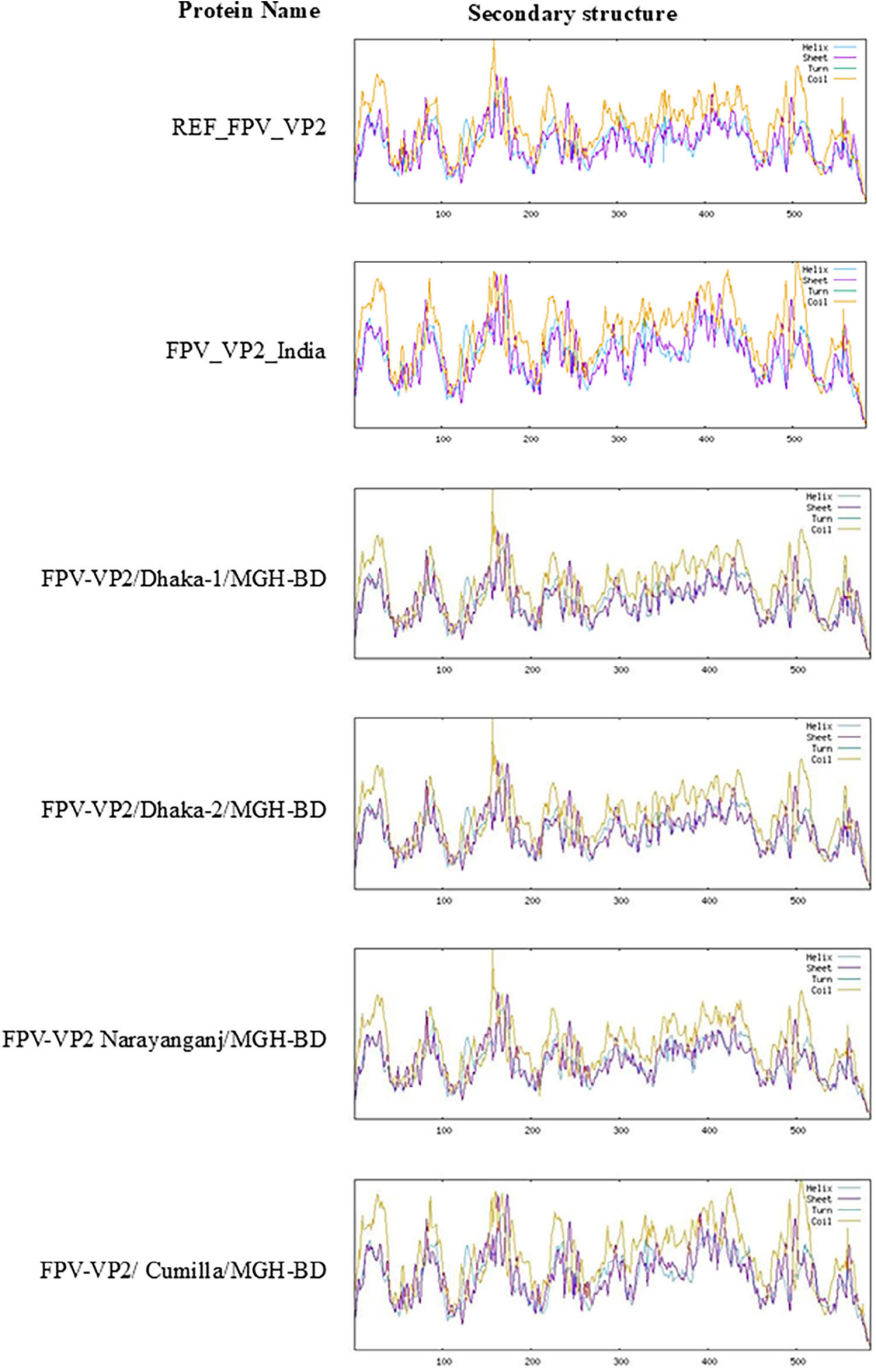
**Secondary structure analysis of the VP2 protein in identified FPV isolates and reference strains**. Secondary structures were predicted and analysed using the SOPMA online server with default parameters.

**FIGURE 9:**
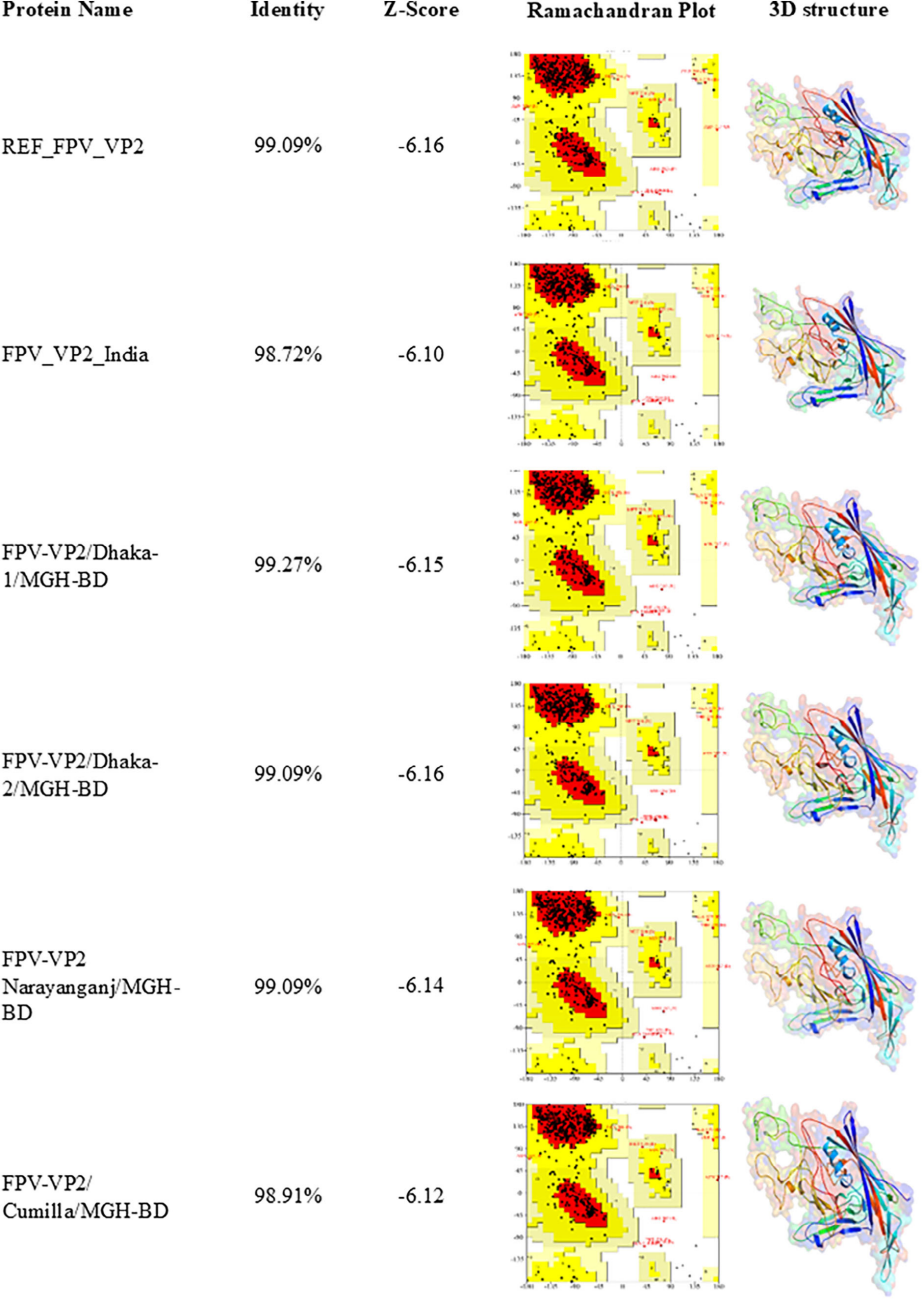
**Prediction and analysis of the tertiary structure of the VP2 protein**. The hydrophobicity of the predicted VP2 protein structures was analysed using SWISS‐MODEL and compared with reference proteins. Identity, *Z*‐score, Ramachandran plots and hydrophobic surface regions (orange patches) are depicted.

**FIGURE 10:**
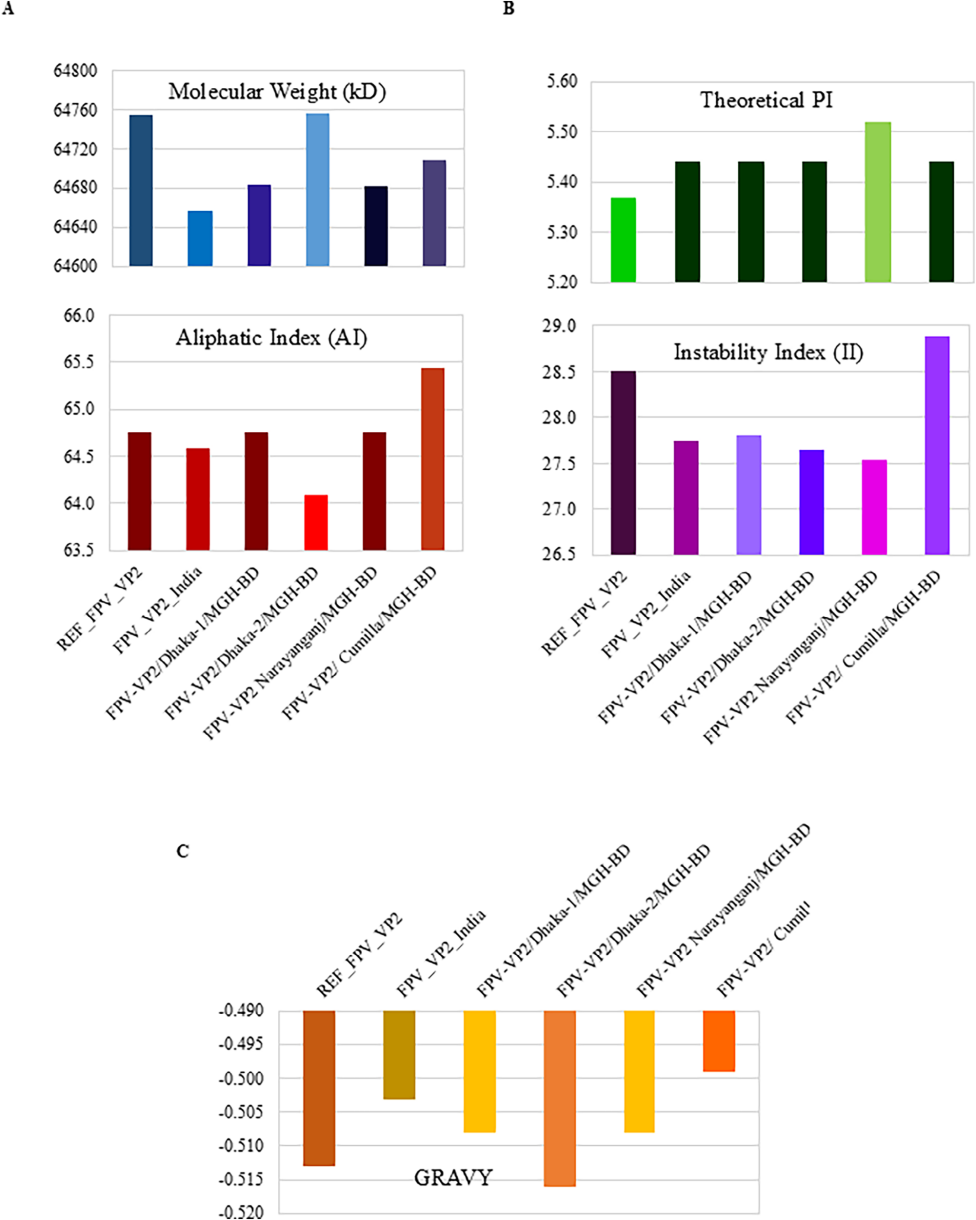
**Physicochemical properties of the VP2 protein compared with reference proteins**. Clustered column bar diagrams depict various properties. (A) Molecular weight and aliphatic index. (B) Theoretical pI and instability index. (C) GRAVY value of the VP2 capsid protein.

The impact of nucleotide substitutions on aa mutations in VP2 proteins was analysed. All VP2 proteins of the identified FPV isolates exhibited an E299G mutation compared to REF_FPV_VP2 (Figure 7). The S122N and T179S mutations were found in three isolates, FPV‐VP2/Dhaka‐1/MGH‐BD, FPV‐VP2/Dhaka‐2/MGH‐BD and FPV‐VP2/Narayanganj/MGH‐BD compared to FPV_VP2_India. Moreover, FPV‐VP2/Cumilla/MGH‐BD displayed two aa mutations at positions T236I and D237E compared to both reference sequences, as well as another substitution at V401I compared to FPV_VP2_India (Figure 7). Additionally, an L111W mutation was reported only in FPV‐VP2/Dhaka‐2/MGH‐BD compared to REF_FPV_VP2 (Figure 7). Secondary structure analysis of VP2 proteins from all identified FPV isolates revealed slight variations in the structures of beta‐turns, random coils, alpha‐helices and extended strands compared to the reference sequences (Figure 8 and Table 3). Despite minor structural inconsistencies, the tertiary structures of the identified FPV isolates were of high quality, as confirmed by the Ramachandran plot and *Z*‐score analysis (Figure 9). Tertiary structural analysis of VP2 proteins from the four identified FPV isolates, in comparison with reference sequences, revealed alterations in their physicochemical characteristics, including molecular weight, theoretical pI, aliphatic index, instability index and GRAVY value (Figure 10). The FPV‐VP2/Dhaka‐1/MGH‐BD, FPV‐VP2/Narayanganj/MGH‐BD and FPV‐VP2/Cumilla/MGH‐BD exhibited similar most‐favoured regions (85%), generously allowed regions (1.50%) and additional allowed regions (12.60%) compared to FPV_VP2_India. However, a slight increase in most‐favoured regions (85.3%) and additional allowed regions (12.8%) was observed in FPV‐VP2/Dhaka‐2/MGH‐BD, with a decrease in generously allowed regions (1.1%) (Table 4).

**TABLE 3:** Secondary structure analysis of VP2 protein compared with reference proteins.

Protein name	Alpha helix	Extended strand	Random coil
REF_FPV_VP2	3.77	18.66	77.57
FPV_VP2_India	4.45	18.49	77.06
FPV‐VP2/Dhaka‐1/MGH‐BD	3.94	19.35	76.71
FPV‐VP2/Dhaka‐2/MGH‐BD	3.94	19.35	76.71
FPV‐VP2/Narayanganj/MGH‐BD	3.60	18.66	77.74
FPV‐VP2/Cumilla/MGH‐BD	4.45	17.81	77.74

Abbreviation: FPV, *Feline panleukopenia* virus.

**TABLE 4:** Protein structure validation.

	Statistics of Ramachandran plot				
Protein name	Most favoured regions (%)	Additional allowed regions (%)	Generously allowed regions (%)	Disallowed regions (%)	*Z*‐score
REF_FPV_VP2	84.2	13.4	1.5	0.9	−6.16
FPV_VP2_India	85	12.6	1.5	0.9	−6.10
FPV‐VP2/Dhaka‐1/MGH‐BD	85	12.6	1.5	0.9	−6.15
FPV‐VP2/Dhaka‐2/MGH‐BD	85.3	12.8	1.1	0.9	−6.16
FPV‐VP2/Narayanganj/MGH‐BD	85	12.6	1.5	0.9	−6.14
FPV‐VP2/Cumilla/MGH‐BD	85	12.6	1.5	0.9	−6.12

Abbreviation: FPV, *Feline panleukopenia* virus.

## 4 Discussion

*F. panleukopenia* is one of the most contagious and fatal diseases affecting the global feline population. Despite routine vaccination practices, the incidence of FPV infection is rising among cats in Bangladesh. FPV detection in suspected cases is commonly performed using rapid antigen test kits; however, these tests may yield false results (Gans et al. 2022; Herbert et al. 2024). Moreover, genomic analyses have revealed unique variations in the antigenicity of FPV field and vaccine strains (Raja et al. 2024). In this study, we conducted a comparative analysis of the sensitivity and specificity of rapid antigen test kits versus PCR for FPV detection. Additionally, we investigated the evolutionary origin of FPV circulating in Bangladesh by analysing *VP2* gene sequences, identifying mutations and predicting structural changes in VP2 proteins of the identified strains.

Detection of FPV using rapid antigen testing and PCR revealed significant differences in sensitivity and specificity. The IC strip test detected FPV in 84% of samples, whereas PCR confirmed only 60% positivity. This discrepancy likely arises due to differences in detection mechanisms. The IC test identifies viral antigens in faecal samples, whereas PCR amplifies viral DNA, ensuring higher specificity (Raheena et al. 2017). False positives in the IC test may result from antigen persistence or cross‐reactivity with closely related viruses, such as CPV (Sykes 2013). Additionally, IC test based diagnostic performance may be compromised by an operational factor, including result variability due to user handling (Miller and Sikes 2015). Hence, the implementation of IC test kits from reputable manufacturers should be accompanied by proper user training, strict adherence to standardized protocols and local validation efforts to ensure reliable detection of FPV in resource‐constrained settings. As PCR targets the *VP2* gene, which encodes the viral capsid protein, it offers greater accuracy due to its conserved nature (Kabir et al. 2023; Xue et al. 2023).

FPV primarily targets rapidly dividing cells, including those in the intestinal crypts and bone marrow, leading to severe immunosuppression and enteritis (Litster and Benjanirut 2014). The clinical symptoms observed in this study, including diarrhoea, vomiting and anorexia, result from the destruction of intestinal epithelial cells, leading to malabsorption and increased gut permeability (Ramadhani et al. 2024). The higher susceptibility observed in male cats aligns with previous studies; however, the underlying cause remains unclear, as this study did not investigate behavioural factors that may influence exposure risk (Rehme et al. 2022). Additionally, the similar infection rates among kittens (≤5 months) and older cats (>5 months) indicate that unvaccinated individuals remain highly vulnerable regardless of age (Rehme et al. 2022). The findings highlight the diagnostic significance of clinical signs such as diarrhoea and foul‐smelling faeces in case of FPV. Diarrhoea results from enterocyte depletion, whereas malodorous faeces may be attributed to dysbiosis and bacterial overgrowth in the gut (Sykes 2013). These findings reinforce the critical role of vaccination, as 92% of infected cats in this study were unvaccinated, confirming its importance in controlling FPV spread. However, although a few vaccinated cats were found positive for FPV, the sample size was too limited to draw any reliable statistical comparison regarding severity between vaccinated and unvaccinated cats. Furthermore, the study was conducted in specific districts which were previously unrepresented in FPV surveillance. These regional data help fill critical epidemiological gaps, thereby strengthening the overall national surveillance framework. By revealing local strain diversity and identifying high‐risk, unvaccinated populations, our findings offer valuable guidance for improving disease control measures and optimizing vaccination programmes across Bangladesh.

Phylogenetic analysis of *VP2* gene sequences revealed that three FPV isolates formed an independent branch closely related to Chinese strains, whereas FPV‐VP2/Cumilla/MGH‐BD shared similarities with strains from both China and Portugal. Although the partial *VP2* gene sequences showed phylogenetic similarity with strains from other countries, the limited number of analysed samples prevents drawing a definitive conclusion about possible cross‐border transmission of FPV into Bangladesh—likely facilitated by international pet trade and animal migration—highlighting the need for more comprehensive regional disease surveillance (Chowdhury et al. 2021). Further investigations should include full‐length VP2 or whole‐genome sequencing from a larger and more diverse sample pool, encompassing a wider range of geographic regions and clinical cases, to enhance the accuracy of phylogeographic mapping and viral evolutionary analysis. The *VP2* gene, crucial for viral infectivity and host adaptation, exhibited multiple mutations across the identified FPV strains (Li et al. 2024). The A756G and A896G substitutions, found in all isolates, suggest ongoing viral evolution and potential adaptation to local feline populations (Wen et al. 2024; Zhang et al. 2024). Mutations such as E299G and S122N may impact capsid stability and receptor‐binding affinity, potentially influencing virulence (Yi et al. 2021). Structural alterations in the VP2 protein, including T236I and D237E mutations, may contribute to changes in antigenicity and immune evasion mechanisms (Leal et al. 2020; Li et al. 2017; Li et al. 2024). Secondary structure analysis revealed subtle shifts in beta‐turns, random coils and alpha‐helices of the VP2 protein, which may affect capsid assembly and stability (Wen et al. 2024; Zhang et al. 2024). Tertiary structural modelling suggested that all identified FPV strains retained overall structural integrity, despite minor physicochemical variations. Changes in molecular weight, theoretical pI and aliphatic index suggest potential implications for host‐virus interactions (Li et al. 2024). However, these interpretations are stated on the basis of previously published literature and still remain speculative, as no functional assays were conducted in this study to confirm such effects.

In conclusion, this study provides critical insights into FPV detection accuracy, epidemiological patterns and genetic evolution in previously unreported regions, thereby expanding the geographic coverage of FPV monitoring in Bangladesh and strengthening the national molecular surveillance framework. The higher specificity of PCR over the IC test highlights the necessity of molecular confirmation in diagnostic workflows. The VP2 mutations and structural variations indicate ongoing potential viral adaptation, necessitating continuous genomic surveillance. Given the high prevalence of FPV in unvaccinated cats, improved vaccination coverage and targeted regional monitoring are essential to mitigate future outbreaks. However, whole‐genome sequencing of both field and vaccine strains is required to better understand the genetic relationship between naturally circulating strains and the vaccine strain.

## Author Contributions

Md. Golzar Hossain designed the study. Nurejunnati Jeba, Md. Masum Billah Tarafder, Mohammad Bayazid Bostami, S. M. Nazmul Hasan and Abdul Mannan collected the samples. Nurejunnati Jeba, Roni Mia, S. M. Nazmul Hasan conducted the laboratory experiments. A. K. M. Anisur Rahman, Nurejunnati Jeba and Roni Mia analysed data. Roni Mia, Raduyan Farazi and Anandha Mozumder analysed the protein structures. Nurejunnati Jeba, Roni Mia and Md. Golzar Hossain wrote the draft version of the manuscript. Sharmin Akter, Sukumar Saha, Tofazzal Islam, Antonio Charlys da Costa, Elcio Leal and Md. Golzar Hossain reviewed and edited the manuscript. All authors have read and agreed to the published version of the manuscript.

## Ethics Statement

All the protocols in this investigation have been performed in accordance with the guidelines of the Animal Welfare and Experimentation Ethics Committee (AWEEC) of Bangladesh Agricultural University.

## Conflicts of Interest

The authors declare no conflicts of interest.

## Peer Review

The peer review history for this article is available at https://publons.com/publon/10.1002/vms3.70594.

## Supporting information




**Table S1**. Clinical and diagnostic data of study subjects based on sample collection locations.

## Data Availability

The data generated in this study are available in the supplementary table and within the manuscript.
