# Supplementary material for: Comparative Detection and Genetic Characterization of Feline Panleukopenia Virus in Bangladesh
Source: Vet Med Sci. 2025 Aug 22;11(5):e70594. doi: 10.1002/vms3.70594 (PMC12372610; doi:10.1002/vms3.70594)
Supplement: Supplementary file 1 — Table S1. Clinical and diagnostic data of study subjects based on sample collection locations. [file VMS3-11-e70594-s001.docx]

**Supplementary table 1. Clinical and diagnostic data of study subjects based on sample collection locations**

| Sample ID | Location | Sex | Age | Vaccination  status | IC test | PCR | Diarrhea | Vomition | Anorexia | Bad odor in feces | Fever | Weakness |
| --- | --- | --- | --- | --- | --- | --- | --- | --- | --- | --- | --- | --- |
| 1 | Narayanganj | Male | 6m | Non Vaccinated | Yes | Yes | yes | Yes | Yes | Yes | No | Yes |
| 2 | Dhaka | Male | 1.5m | Non Vaccinated | Yes | Yes | yes | Yes | Yes | No | No | Yes |
| 3 | Dhaka | Female | 4m | Non Vaccinated | Yes | Yes | yes | Yes | Yes | Yes | Yes | Yes |
| 4 | Dhaka | Female | 4m | Non Vaccinated | Yes | No | yes | Yes | Yes | No | No | Yes |
| 5 | Dhaka | Male | 6m | Non Vaccinated | Yes | Yes | yes | Yes | Yes | Yes | Yes | No |
| 6 | Dhaka | Male | 8m | Non Vaccinated | Yes | Yes | yes | Yes | Yes | No | Yes | Yes |
| 7 | Narayanganj | Female | 12m | Non Vaccinated | Yes | Yes | yes | Yes | Yes | Yes | No | Yes |
| 8 | Dhaka | Male | 3m | Non Vaccinated | No | No | yes | Yes | Yes | No | No | No |
| 9 | Dhaka | Female | 4m | Non Vaccinated | No | No | No | No | Yes | No | Yes | No |
| 10 | Dhaka | Female | 30m | Non Vaccinated | Yes | No | No | No | Yes | Yes | No | No |
| 11 | Dhaka | Male | 24m | Non Vaccinated | Yes | No | No | Yes | Yes | No | No | No |
| 12 | Narayanganj | Female | 12m | Non Vaccinated | Yes | Yes | yes | Yes | Yes | Yes | No | Yes |
| 13 | Dhaka | Female | 3m | Non Vaccinated | Yes | Yes | No | Yes | Yes | No | No | No |
| 14 | Dhaka | Male | 2m | Non Vaccinated | Yes | Yes | yes | Yes | Yes | Yes | No | Yes |
| 15 | Narayanganj | Male | 3m | Non Vaccinated | Yes | Yes | yes | No | Yes | No | Yes | No |
| 16 | Cumilla | Male | 2m | Non Vaccinated | Yes | Yes | yes | No | Yes | Yes | Yes | Yes |
| 17 | Dhaka | Male | 2m | Non Vaccinated | No | No | No | Yes | No | No | No | No |
| 18 | Dhaka | Male | 3m | Vaccinated | Yes | Yes | yes | Yes | Yes | No | No | Yes |
| 19 | Dhaka | Male | 11m | Non Vaccinated | No | No | No | Yes | Yes | No | No | Yes |
| 20 | Dhaka | Male | 84m | Vaccinated | Yes | No | yes | Yes | Yes | No | No | No |
| 21 | Narayanganj | Male | 4m | Non Vaccinated | Yes | No | yes | Yes | Yes | No | No | Yes |
| 22 | Narayanganj | Male | 3m | Non Vaccinated | Yes | No | No | Yes | Yes | No | Yes | No |
| 23 | Narsingdi | Male | 8m | Non Vaccinated | Yes | Yes | yes | Yes | Yes | Yes | No | No |
| 24 | Narayanganj | Male | 6m | Non Vaccinated | Yes | Yes | yes | Yes | Yes | Yes | No | Yes |
| 25 | Dhaka | Male | 1.5m | Non Vaccinated | Yes | Yes | yes | Yes | Yes | Yes | No | Yes |
| 26 | Dhaka | Female | 5m | Non Vaccinated | No | No | yes | Yes | No | No | No | No |
| 27 | Dhaka | Male | 36m | Non Vaccinated | Yes | No | yes | Yes | Yes | No | No | Yes |
| 28 | Dhaka | Male | 24m | Vaccinated | Yes | No | No | Yes | Yes | No | No | No |
| 29 | Dhaka | Male | 3m | Non Vaccinated | Yes | Yes | yes | Yes | Yes | Yes | Yes | Yes |
| 30 | Dhaka | Female | 18m | Non Vaccinated | Yes | No | No | Yes | Yes | No | No | No |
| 31 | Dhaka | Male | 3m | Vaccinated | Yes | Yes | yes | Yes | Yes | No | No | Yes |
| 32 | Dhaka | Female | 5m | Non Vaccinated | Yes | No | No | Yes | Yes | No | Yes | Yes |
| 33 | Narsingdi | Male | 9m | Non Vaccinated | Yes | Yes | yes | Yes | Yes | Yes | No | Yes |
| 34 | Narayanganj | Male | 5.5m | Non Vaccinated | Yes | No | yes | Yes | Yes | No | No | No |
| 35 | Dhaka | Male | 2.5m | Non Vaccinated | No | No | No | Yes | No | No | Yes | No |
| 36 | Narayanganj | Female | 11m | Non Vaccinated | Yes | Yes | yes | Yes | Yes | Yes | No | Yes |
| 37 | Narayanganj | Male | 6m | Non Vaccinated | Yes | Yes | yes | Yes | Yes | No | Yes | Yes |
| 38 | Dhaka | Male | 7m | Non Vaccinated | Yes | Yes | yes | Yes | Yes | Yes | No | Yes |
| 39 | Dhaka | Male | 2m | Non Vaccinated | No | No | yes | No | Yes | No | No | Yes |
| 40 | Cumilla | Male | 3m | Non Vaccinated | Yes | Yes | yes | Yes | Yes | Yes | Yes | No |
| 41 | Dhaka | Female | 6m | Non Vaccinated | Yes | Yes | yes | Yes | Yes | Yes | No | Yes |
| 42 | Dhaka | Male | 10m | Non Vaccinated | No | No | yes | Yes | Yes | No | Yes | No |
| 43 | Dhaka | Male | 5m | Non Vaccinated | Yes | Yes | yes | Yes | Yes | Yes | No | Yes |
| 44 | Narayanganj | Male | 5m | Non Vaccinated | Yes | Yes | yes | Yes | Yes | No | No | Yes |
| 45 | Narayanganj | Male | 3m | Non Vaccinated | Yes | No | yes | No | Yes | No | No | No |
| 46 | Narayanganj | Female | 12m | Non Vaccinated | Yes | Yes | yes | Yes | Yes | Yes | No | No |
| 47 | Dhaka | Male | 2.5m | Non Vaccinated | Yes | Yes | yes | Yes | No | No | No | No |
| 48 | Narayanganj | Male | 3m | Non Vaccinated | Yes | Yes | No | Yes | Yes | No | No | No |
| 49 | Dhaka | Male | 2m | Non Vaccinated | Yes | Yes | yes | Yes | No | No | No | Yes |
| 50 | Dhaka | Female | 3m | Non Vaccinated | Yes | Yes | yes | Yes | Yes | Yes | No | No |
